# Acute Myeloid Leukemia Chemo-Resistance Is Mediated by E-selectin Receptor CD162 in Bone Marrow Niches

**DOI:** 10.3389/fcell.2020.00668

**Published:** 2020-07-24

**Authors:** Johanna Erbani, Joshua Tay, Valerie Barbier, Jean-Pierre Levesque, Ingrid G. Winkler

**Affiliations:** Mater Research Institute – The University of Queensland, Translational Research Institute, Woolloongabba, QL, Australia

**Keywords:** acute myeloid leukemia, bone marrow niches, E-selectin, PSGL-1 (CD162), adhesion, chemoresistance

## Abstract

The interactions of leukemia cells with the bone marrow (BM) microenvironment is critical for disease progression and resistance to treatment. We have recently found that the vascular adhesion molecule E-(endothelial)-selectin is a key niche component that directly mediates acute myeloid leukemia (AML) chemo-resistance, revealing E-selectin as a promising therapeutic target. To understand how E-selectin promotes AML survival, we investigated the potential receptors on AML cells involved in E-selectin-mediated chemo-resistance. Using CRISPR-Cas9 gene editing to selectively suppress canonical E-selectin receptors CD44 or P-selectin glycoprotein ligand-1 (PSGL-1/CD162) from human AML cell line KG1a, we show that CD162, but not CD44, is necessary for E-selectin-mediated chemo-resistance *in vitro*. Using preclinical models of murine AML, we then demonstrate that absence of CD162 on AML cell surface leads to a significant delay in the onset of leukemia and a significant increase in sensitivity to chemotherapy *in vivo* associated with a more rapid *in vivo* proliferation compared to wild-type AML and a lower BM retention. Together, these data reveal for the first time that CD162 is a key AML cell surface receptor involved in AML progression, BM retention and chemo-resistance. These findings highlight specific blockade of AML cell surface CD162 as a potential novel niche-based strategy to improve the efficacy of AML therapy.

## Introduction

Acute myeloid leukemia (AML) is an aggressive hematological cancer, where immature myeloid blasts accumulate within the bone marrow (BM) and perturb normal hematopoiesis (DiNardo and Cortes, 2016). Prognosis is poor, with 60–80% of adult AML patients succumbing from disease relapse and a median overall survival of 15 months (Australian Institute of Health and Welfare, 2010; Cogle et al., 2016; DiNardo and Cortes, 2016). Standard therapy for AML has not changed in over 30 years (Dombret and Gardin, 2016), highlighting the difficulty in improving therapeutic options with the current research approaches.

While most studies have focused on targeting the intrinsic properties of AML cells that lead to treatment resistance, extrinsic factors can also play a significant role in leukemia development and response to treatment. In recent years, the contribution of the BM microenvironment (niche) has been under intense investigation and is a potentially new approach for discovery of novel therapeutic targets (Lane et al., 2009; Tay et al., 2017; Duarte et al., 2018).

The vascular adhesion molecule E-(endothelial)-selectin is exclusively expressed on activated endothelial cells, and has been shown to be a key regulator of hematopoietic stem cell (HSC) activity within the BM vascular niche (Winkler et al., 2012). Using mice knocked-out for the E-selectin gene and a therapeutically relevant E-selectin antagonist (GMI-1271/Uproleselan), we have recently discovered that E-selectin also regulates malignant AML cells by influencing therapy response. Indeed, the absence or blockade of E-selectin at the vascular niche sensitizes AML blasts to chemotherapy and significantly extends disease-free survival duration in treated leukemic mice (Barbier et al., 2020). These findings highlight endothelial E-selectin as a promising therapeutic target, and are the basis for two phase III clinical trials to test the activity of GMI-1271 in combination with chemotherapy in adult AML patients (NCT03616470, NCT03701308).

In this current study, we investigate the contribution of the canonical E-selectin receptors in mediating AML chemo-resistance.

Selectins are cell adhesion molecules involved in leukocyte (and platelet) homing and recruitment to sites of inflammation (Ley, 2003; Muller, 2011; Sreeramkumar et al., 2013). Two well-characterized (canonical) E-selectin receptors are involved in this process: CD162, also called P-selectin glycoprotein ligand-1 (PSGL-1) as well as a specific sialofucosylated glycoform of CD44 called hematopoietic cell E-/L-selectin ligand (HCELL) (Dimitroff et al., 2001, 2005; Merzaban et al., 2011; Zarbock et al., 2011). CD44 and CD162 have been extensively studied in their role as E-selectin receptors. They are constitutively expressed by neutrophils and have been described as E-selectin ligands on other leukocytes such as T-cells, monocytes and human hematopoietic progenitor cells (Asa et al., 1995; Moore, 1998; Xie et al., 1999; Hirata et al., 2000; Dimitroff et al., 2001; Kieffer et al., 2001; Katayama et al., 2005; Martinez et al., 2005; Hidalgo et al., 2007; Nácher et al., 2011; Silva et al., 2017; Videira et al., 2018).

A range of other factors have additionally been reported to bind to E-selectin, such as E-selectin ligand-1 (ESL-1) on mouse but not human neutrophils (Steegmaler et al., 1995; Hidalgo et al., 2007; Sreeramkumar et al., 2013); L-selectin on human but not mouse neutrophils and lymphocytes (Jones et al., 1997; Zöllner et al., 1997); CD43 on human and mouse lymphocytes, mononuclear cells and neutrophils (Xia et al., 2004; Matsumoto et al., 2005; Alcaide et al., 2007; Nonomura et al., 2008; Merzaban et al., 2011; Silva et al., 2017); endoglycan on human B cells (Kerr et al., 2008); death receptor-3 on colon cancer cells (Gout et al., 2006); and sialofucosylated membrane glycolipids on human neutrophils and HSCs (Nimrichter et al., 2008; Winkler et al., 2012).

Unlike the other known E-selectin ligands, both CD44 and CD162 receptors have been reported in both mouse and human hematopoietic cells, and have well defined roles in facilitating the rolling of mature leukocytes and/or hematopoietic stem and progenitor cells (HSPCs) on activated E-selectin-expressing vasculature, as well as HSPC homing to the BM (Dimitroff et al., 2001; Katayama et al., 2003; Sackstein, 2004; Winkler et al., 2004; Merzaban et al., 2011). Of note, E-selectin is not the only selectin expressed on activated endothelium. P-(platelet and endothelium)-selectin also plays an important role in leukocyte homing (Nabors et al., 2013). Our previous data suggests that E-selectin may play a greater role in cell signaling, whereas P-selectin is more important for homing (Winkler et al., 2012; Nabors et al., 2013; Barbier et al., 2020). Consistent with this notion, some leukocyte receptors, specifically CD162, may also bind both endothelial selectins (Snapp et al., 1998; Winkler et al., 2004).

We have previously found that when E-selectin becomes expressed at the vascular HSC niche in the BM, it directly awakens otherwise dormant HSC into commitment and differentiation, at the expense of self-renewal (Winkler et al., 2012). In these experiments neither CD162 or CD44 appeared to play a role in E-selectin-mediated adhesion or regulation of HSCs at the BM vascular niche (Winkler et al., 2012).

Herein, we now explore the hypothesis that AML may utilize a distinct E-selectin receptor repertoire compared to endogenous HSC, or at least that the glycosylation levels of receptor backbones may differ with oncogenic transformation. Indeed, aberrant and overall increased level of glycosylation is recognized as one of the hallmarks of oncogenic transformation (Hakomori, 1996), and can result in increased or a *de novo* ability of malignant cells to bind biological lectins like E-selectin. Thus, herein we investigate the functional roles of CD44 and CD162 as E-selectin ligands on AML cells in human and preclinical mouse models. We show that CD162, but not CD44, is required for E-selectin-mediated chemo-resistance *in vitro* and report a novel role for CD162 in AML progression and response to treatment *in vivo*.

## Materials and Methods

### Mice and Tissue Processing

All experiments on mice were approved by the Animal Ethics committee of the University of Queensland. All mice used were sex- and age-matched between 7–12 weeks. Generally, donors were males, and recipients for long-term survival experiments were females. C57BL/6 and B6.SJKL CD45.1^+^ congenic mice were purchased from the Animal Resource Center (Perth, Australia). *Selplg*^–/–^ mice were kindly provided by B. Furie (Beth Israel Deaconess Medical Center, Harvard Medical School, Boston) and had been backcrossed over 10 times into the C57BL/6 background. Mice were housed in Techniplast Greenline IVC caging in a room with controlled temperature (22–26 degrees Celsius) and humidity (30–70%).

For BM collection, femurs were flushed with a 23G needle in 1 mL ice-cold phosphate buffered saline (PBS, Thermo Fisher) containing 2% newborn calf serum (NCS, Thermo Fisher). The leukocyte content of the samples was then counted on an AcT Diff Coulter Counter (Beckman Coulter).

For test bleeds, heparinized blood was collected from the lateral tail vein and red blood cells were lysed in 0.15 M NH_4_Cl, 10 mM NaHCO3, pH 7.4 lysis buffer. Lysed samples were stained with 2 μg/ml 7-aminoactinomycin D (7-AAD) to exclude dead cells. The presence of GFP^+^ AML cells in the blood was assessed by flow cytometry using the CytoFLEX S analyzer (Beckman Coulter).

Calculation of whole-body AML blast numbers in BM and blood for Figure 4E were performed as previously described (Winkler et al., 2013). In summary one femur represents 5.6% total BM, and total blood volume of a mouse was calculated as 0.8 mL peripheral blood per 10 g body weight.

### Generation of AML From WT and *Selplg*^–/–^ Murine HSPCs

GPE86 packaging cells producing MLL-AF9-containing expression retroviruses (following transfection with MSCV-MLL-AF9-IRES-GFP plasmid) (Zuber et al., 2009) were irradiated at 15 Gy. Fetal liver cells collected from C57BL/6 or *Selplg*^–/–^ embryos at E13 or E14 were incubated overnight with the irradiated packaging cells in fresh Dulbecco Modified Eagle’s medium (DMEM, Thermo Fisher) containing 10% fetal calf serum (FCS, Thermo Fisher), 10 ng/mL recombinant human (rh) IL-11 (R&D System), 10 ng/mL recombinant murine (rm) thrombopoietin, 50 ng/mL rm stem cell factor (SCF) (both from Peprotech) and 5 μg/mL polybrene (Sigma-Aldrich). The transduced fetal liver cells were then harvested, washed in medium and retro-orbitally injected into 4Gy irradiated C57BL/6 recipients.

Emergence of AML in recipient mice was monitored by flow cytometry from regular tail vein test-bleeds for appearance of GFP^+^ AML cells in the circulation. Once over 40% of peripheral blood leukocytes were GFP^+^ (typically 30–60 days post transplantation) mice were euthanized. Femora, tibiae, hips, and spine were removed and crushed in ice-cold PBS with 2% NCS using a mortar and pestle, then filtered through a 40 μm cell strainer. BM KIT^+^ AML blasts were further enriched from this cell suspension using mouse CD117-conjugated Magnetic Activated Cell Sorting (MACS) beads and “POSSEL” positive selection on auto-MACS (Miltenyi, Germany) as previously described (Winkler et al., 2012).

### Flow Cytometry and Stains

All antibodies used are listed in Supplementary Table 1. Stained samples were acquired on a CytoFLEX S analyzer (Beckman Coulter), and analysis was performed using FlowJo v10 (TreeStar).

#### E-Selectin Binding

For analysis of E-selectin binding potential, recombinant E-selectin–human-IgM fusion protein supernatants or matching empty-vector human-IgM as negative control (plasmids kindly donated by Karen Snapp, University of Illinois, IL, United States) were precomplexed with AlexaFluor647-conjugated donkey F(ab)_2_ antibody to human IgM (Jackson Immunoresearch) for 2 h at room temperature, as previously described (Katayama et al., 2005). This construct was then added to AML blasts (either BM cells from leukemic mice or KG1a cells) pre-stained with cell surface markers when specified. After 30 min on ice, cells were washed in X-VIVO 15 media (Lonza) and analyzed by flow cytometry with 2 μg/mL 7-AAD to gate on viable cells. Two negative (non-binding) controls were included, (i) an identical parallel stain in presence of 15 mM EDTA (which is ∼4 mM in excess of the metallic divalent cations in the media used, as E-selectin binding is strictly Ca^2+^-dependent), and (ii) a parallel stain with AF647-labeled empty-vector instead of recombinant E-selectin was also used.

#### Cell Cycle Analysis

B6.SJL mice (CD45.1^+^) mice were transplanted with 10^4^ BM GFP^+^KIT^+^ leukemic blasts generated from transduced C57BL/6 (CD45.2^+^) WT or *Selplg*^–/–^ HSCs. The use of a CD45.1^+^ recipients were required in this experiment as GFP fluorescence (our usual marker for transduced AML) is lost after fixation. Recipients of WT AML blasts were transplanted ∼4 weeks before euthanasia, and recipients of *Selplg*^–/–^ AML blasts ∼12 weeks before euthanasia. Both cohorts were euthanized on the same day.

One million whole BM cells were stained with anti-CD45.2-FITC and anti-KIT-APC. Cells were then fixed and permeabilized using the FIX and PERM^TM^ Cell Permeabilization Kit (Thermo Fisher) before staining with monoclonal antibody to Ki67-AF700 then 25 μM Hoescht33342.

### Imaging Flow Cytometry

For E-selectin receptors co-localization assays, KG1a cells (from ATCC CCL-246.1) were incubated with the preformed complex of E-selectin–IgM (or matching empty vector) and AF647-labeled antibody to IgM (as described above), in a microfuge tube at 37°C with gentle agitation. After 20 min of incubation, tubes were placed on ice and cells washed in X-VIVO media, before staining with human CD44-PECy7 and CD162-BV421 conjugated antibody. Stained cells were then analyzed for receptor co-localization by imaging flow cytometry with Amnis^®^ ImageStream^®^X (EMD Millipore) at 60X magnification. Analysis of receptor co-localization and polarization was performed using IDEAS^®^ software (EMD Millipore).

### *In vitro* Chemo-Sensitivity Assay

Tissue culture wells (96-well plates) were coated with recombinant human PECAM-1/CD31, P-selectin, E-selectin and CD14 (non-adhesion control) human IgG1 Fc fusion proteins (purchased from R&D Systems) at a concentration of 5 μg/mL in 30 μL of Tris–HCl 20 mM pH 8.6, overnight at 4°C. Non-coated control wells were also included. The next day, unbound proteins were removed and the wells were washed with PBS before being blocked for one hour in X-VIVO media at 37°C. Ten thousand KG1a cells were then added into each well, in a volume of 100 μL of X-VIVO. The plate was incubated for 7 h at 37°C before Ara-C or saline control was added in 20 μL at final concentration of 10 μg/mL, 100 μg/mL, or 1 mg/mL. After 48 h of incubation at 37°C, cell viability was assessed using the CellTox Green Cytotoxicity assay and CellTiter-Glo 2.0 viability assay (both from Promega) on a PHERAstar microplate reader (BMG Labtech) according to the manufacturer’s instructions.

Two negative (non-adhesion) controls were included for chemo-sensitivity assay, (1) parallel blocked non-coated wells, and (2) parallel control wells coated with recombinant human CD14 IgG1 Fc fusion protein (as non-adhesion control).

### Generation of Receptor Deleted Human AML Cell Line by CRISPR Gene Editing

CRISPR-Cas9 gene editing was used to delete *CD44* and *SELPLG* genes from KG1a cells. Design of the single guide (sg) RNAs and optimization were based on a protocol developed by Charles C. Bell in Andrew Perkins group (Bell et al., 2014) (ACBD, Monash University, Australia) as well as published protocol by Ran et al. (2013).

**FIGURE 1:**
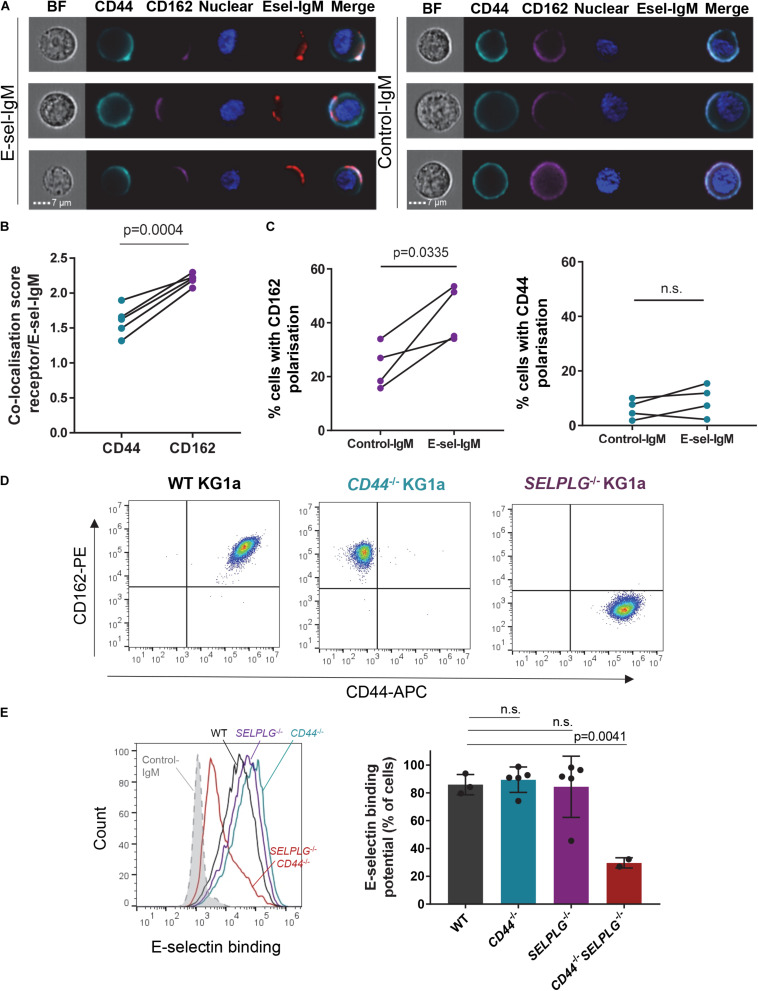
CD44 and CD162 co-localize with E-selectin on human AML cell surface and are redundant for E-selectin binding. **(A–C)** KG1a cells were incubated with a preformed complex of human E-selectin–IgM (or empty-human IgM construct as a negative control) and AF647-labeled antibody to human IgM (red), then stained for E-selectin receptors CD44 (cyan) and CD162 (magenta). **(A)** AMNIS^®^ ImageStream^®^ flow cytometry imaging showing co-localization of receptors with E-selectin-IgM (Esel-IgM). BF: brightfield; Nuclear: Hoechst33342 nuclear stain. **(B)** Quantification of the similarity (co-localization) score. Data shown as paired samples from five independent experiments; each dot represents data from one experiment with *n* = 10,000 individual cells analyzed in each sample from each experiment. Statistical significance was calculated by paired two-tailed *t*-test. **(C)** Percentage of KG1a cells in which CD162 (left panel) or CD44 (right panel) are polarized to one side of the cell surface (receptor distribution restricted to <30% of overall cell surface), as determined by AMNIS^®^ ImageStream^®^ analysis (IDEAS software). Data shown as paired samples from 4 independent experiments; each dot represents data from one experiment with *n* = 10,000 individual cells analyzed in each sample from each experiment. Statistical significance was calculated by paired two-tailed *t*-test. **(D)** Flow cytometry dot plots showing CD44 and CD162 expression on KG1a cell surface, before (WT KG1a) or after (*CD44*^– /–^ and *SELPLG*^– /–^ KG1a) CRISPR-Cas9 gene editing. Cells were gated on singlets/live cells. **(E)** Effect of *CD44* and *SELPLG* gene deletion on KG1a binding to recombinant E-selectin in suspension. To determine E-selectin binding potential, KG1a cells were incubated with a preformed complex of E-selectin–IgM and AF647-labeled antibody to IgM. Left panel: overlays of E-selectin binding to gated singlets/live cells. Dotted gray line: control-IgM negative control; black: WT KG1a; blue: *CD44*^– /–^ KG1a; purple: *SELPLG*^– /–^ KG1a; red: *CD44*^– /–^ *SELPLG*^– /–^ KG1a. An additional negative control was performed by addition of 15mM EDTA to the E-selectin-IgM stain (as E-selectin binding is calcium-dependent) (Supplementary Figure 1). Right panel: Histogram quantifying the percentage of gated cells that bound E-selectin–IgM. *n* = 3, 5, 5, and 2 clones per group for WT, *CD44*^– /–^, *SELPLG*^– /–^ and *CD44*^– /–^ *SELPLG*^– /–^, respectively. Graph shows mean ± SD. Statistical significance was calculated by one-way ANOVA with Dunnett’s correction for multiple comparison.

Deskgen’s CRISPR guide RNA design tool^1^ was used to generate single guide (sg) RNAs sequences with minimal exonic off-target sites. Two sgRNAs were designed for both *CD44* and *SELPLG* (each targeting a different exon), with target sequences as follows: 5′-TCGCTACAGCATCTCTCGGA and 5′-TGGGTTCATAGAAGGGCACG for *CD44*; 5′-ATCTAGG TACTCATATTCGG and 5′-CTCCGGTCCCGGGCAAGCAG for *SELPLG.* The sgRNAs were cloned into the pSpCas9(BB)-2A-GFP (PX458) plasmid (Addgene cat# 48138) after Bpil digestion. Purified plasmids were transfected in KG1a cells using the 4D-Nucleofector^TM^ (Lonza) according to the manufacturer’s instructions with the DI-100 setting. At day 6 post nucleotransfection, cell surface staining (using anti-human conjugated antibodies CD44-APC and CD162-PE, see Supplementary Table 1) revealed the appearance of a small population of cells that were negative for either CD44 or CD162. The next day, single cells from these populations were sorted using BD FACSAria^TM^ Fusion (BD Biosciences) into 96-well plates and cultured in MEMα supplemented with 10% FCS and 1X penicillin-streptomycin-glutamine (all from Gibco). Around 4 weeks later, several KG1a clones emerged from these single cells and were again tested by flow cytometry for cell surface expression of CD44 and CD162. Clones that displayed >99% cells negative for CD44 or CD162 (as shown in Figure 1D) were selected and expanded for use in *in vitro* studies. Control WT KG1a cells were subjected to the same process as transfected KG1a cells except for the omission of sgRNA.

### *In vivo* Chemo-Sensitivity Assay

Cohorts of 4-Gy conditioned C57BL/6 mice were retro-orbitally transplanted with 30,000 WT or *Selplg*^–/–^ AML blasts. Leukemia progression was measured by regular monitoring of mouse wellbeing and blood tests for emergence of GFP^+^ AML cells. Mice with advanced AML (5–40% GFP^+^ AML blasts in blood) were administered two high dose rounds of cytarabine (900 mg/kg intraperitoneally) or saline at 24 and 12 h prior to euthanasia. Femurs of each group were collected, pooled and crushed in PBS with 2% FCS. The equivalent of 1% of a femur was transplanted back into cohorts of 4-Gy conditioned C57BL/6 recipients, and mice were then monitored for wellbeing and duration to relapse (defined as >30% GFP^+^ AML blasts in blood, >80% in the BM and/or signs of morbidity associated with leukemia). Median disease-free survival duration was analyzed by Kaplan Meier survival analysis.

### Statistical Analysis

Prism software version 7 (GraphPad) was used for all statistical analyses. Unless otherwise indicated, one-way ANOVA with Dunnett’s correction was used to compare three or more groups. Statistical differences between two groups were tested by paired or unpaired two-tailed *t*-test as specified. Data was tested for parametric Gaussian distribution using the D’Agostino-Pearson omnibus normality test. Survival curves were plotted using Kaplan-Meier estimates and compared using a Log-rank (Mantel-Cox) test. All data were plotted as mean ± standard deviation (SD) unless otherwise specified.

## Results

### CD162 Co-localizes With E-Selectin Binding Site on Human AML Cell Surface, but Is Redundant for E-Selectin Adhesion

To assess whether canonical receptors CD44 and CD162 played a role in AML binding to E-selectin, we first investigated for their co-localization with surface-bound recombinant E-selectin using human AML CD34^+^ cell line KG1a. With imaging flow cytometry, we observed that both cell surface CD44 and CD162 localized around the E-selectin-IgM-binding site (Figure 1A, left panel). Data for 10,000 AML cells were then objectively quantified using the inbuilt AMNIS^®^ analysis (IDEAS^®^) software and revealed a significantly higher co-localization score (*p* = 0.0004) between CD162 and E-selectin compared to CD44 and E-selectin (Figure 1B).

This analysis also revealed there were intrinsic differences in distribution on cell surface between these two proteins prior to E-selectin contact. Although CD44 was mostly distributed evenly over the entire cell surface, CD162 seemed more likely to be polarized on one side of the cell (Figure 1C). Indeed, overall clustering of CD162 (over less than one third of cell surface) occurred in around 25% of KG1a cells (Figure 1C, left panel). In contrast such “polarization” of CD44 was rare (around 5%) (Figure 1C, right panel). Notably, upon addition of E-selectin-IgM, a stronger clustering of CD162 at the site of E-selectin-IgM binding was observed, with a further significant increase in CD162 polarization (Figure 1C, left panel). By contrast, CD44 polarization was not altered after addition of E-selectin-IgM (Figure 1C, right panel). Together these data suggest that AML cell adhesion to E-selectin may trigger a strong polarization or clustering of CD162 toward the E-selectin-IgM binding site, which was not observed with CD44. As CD162’s cytoplasmic tail is known to bind to, and activate, multiple kinases (Urzainqui et al., 2002; Carlow et al., 2009; Spertini et al., 2012), it is possible that this clustering associates with intracellular signaling.

To determine whether CD44 and CD162 were essential for binding of human AML cells to E-selectin, CRISPR-Cas9 gene editing was used to selectively suppress CD44 and/or CD162 genes (*CD44* and *SELPLG*, respectively) from KG1a cells (representative cell surface CD44 and CD162 staining profiles after Crispr-Cas9 deletion shown in Figure 1D). Surprisingly, absence of neither CD44 nor CD162 alone affected KG1a cell binding to soluble E-selectin-IgM (Figure 1E). However, loss of both receptors led to a significant 65 ± 4.2% drop in E-selectin-IgM binding potential (Figure 1E, right panel). These data suggest that CD162 and CD44 are both important and redundant receptors for E-selectin binding in KG1a cells, and that KG1a also express additional E-selectin receptors.

### CD162 Is Critical for E-Selectin-Mediated Chemo-Resistance of Human AML *in vitro*

**FIGURE 2:**
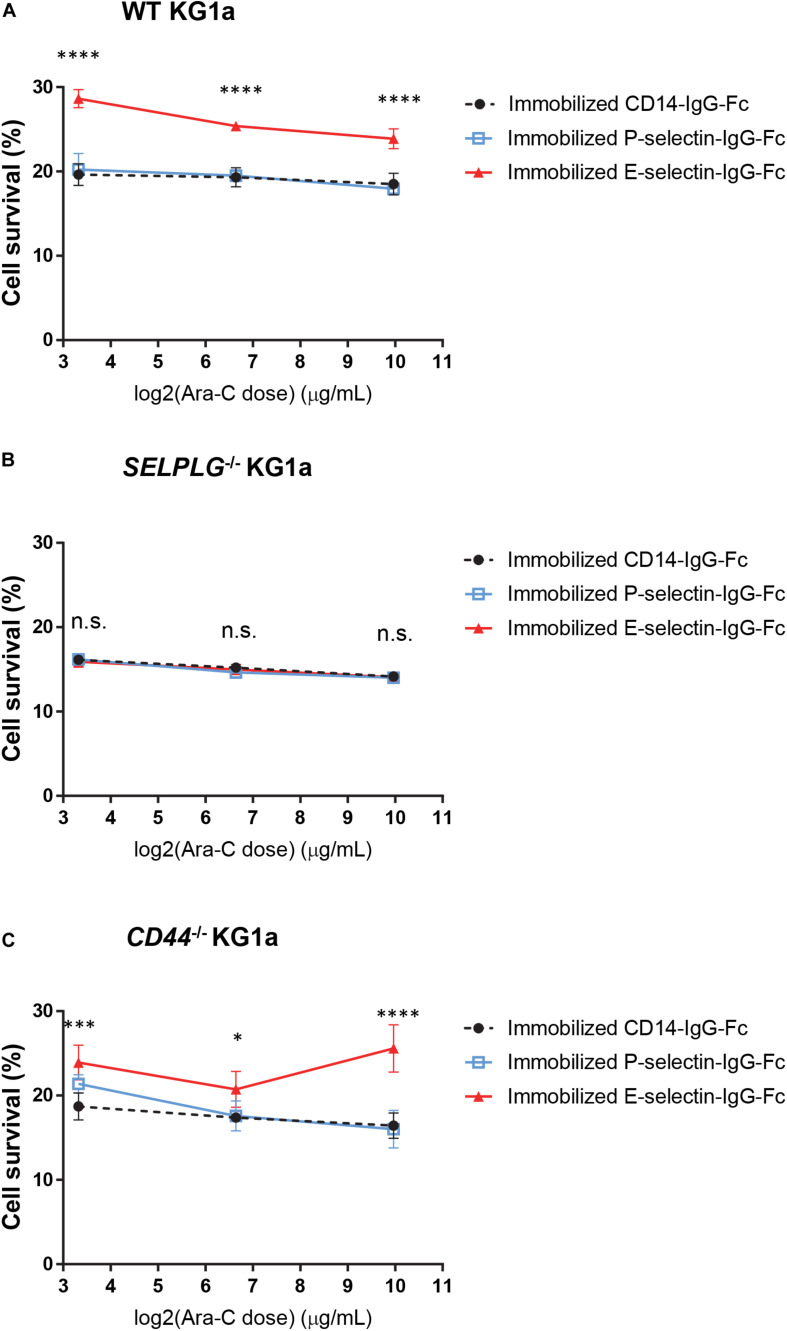
CD162 is important for E-selectin-mediated chemo-resistance *in vitro*. **(A)** WT, **(B)** *SELPLG*^– /–^, and (**C**) *CD44*^– /–^ KG1a cells were immobilized on recombinant adhesion molecules: E-selectin, P-selectin and non-adhesion control CD14, then treated with 10 μg/mL, 100 μg/mL, or 1 mg/mL cytarabine. Cell viability was assessed after 48 h of incubation at 37°C. The percentage of surviving cells was calculated in comparison to untreated cells seeded at the same density and in the same conditions. Each dot represents the average data from five replicate wells. Statistical significance was calculated by one-way ANOVA with Dunnet’s multiple comparison test. ^****^p < 0.0001; ^∗∗∗^*p* < 0.001; ^∗^*p* < 0.05.

We have recently shown that adhesion to E-selectin can directly promote significant resistance to cytotoxic chemotherapy *in vitro* and *in vivo* in mouse models of AML driven by the MLL-AF9 oncogene (Barbier et al., 2020). To determine whether CD44 or CD162 are the potential receptors mediating this chemo-resistance upon E-selectin adhesion, a number of individually derived WT, *CD44*^–/–^, and *SELPLG*^–/–^ KG1a cell lines were adhered to wells coated with three different endothelial cell adhesion molecules (E-selectin, P-selectin and PECAM-1/CD31 or CD14-Fc non-binding control). Cell survival was assessed 48 h after addition of various doses of the chemotherapeutic drug cytarabine (from 10 μg/mL to 1 mg/mL). We found that adhesion to E-selectin significantly increased the survival of WT KG1a to cytarabine at all three doses tested compared to CD14-IgG-Fc non-adhesion control (Figure 2A, *p* < 0.0001), as anticipated from our previous findings using murine AML model (Barbier et al., 2020). When repeated using KG1a cells lacking CD162 (*SELPLG*^–/–^), E-selectin-mediated chemoresistance was no longer observed (Figure 2B). In contrast, when CD44 was deleted, E-selectin-mediated chemo-resistance was again recorded (Figure 2C). None of the other adhesion molecules tested promoted AML cell survival (Figure 2A and Supplementary Figure 2) confirming the specificity of E-selectin as an adhesion molecule promoting survival signaling in human AML cell lines *in vitro.* Together these data suggest the expression of CD162 on AML cells is important for E-selectin-mediated chemo-resistance in our *in vitro* model – providing the first evidence of a potential role for CD162 in mediating AML chemo-resistance.

### CD162 Expression on Murine AML *in vivo* Is Important for E-Selectin Binding, BM Niche Retention and Leukemia Progression

For *in vivo* confirmation, we generated murine AML by retroviral transduction of fetal liver cells from either *Selplg*^–/–^ (CD162^–/–^) or WT mice with a bicistronic expression vector containing GFP and MLL-AF9 (11q23-rearrangement mimic) fusion oncogene as previously described (Barbier et al., 2020). Once established in primary mice, BM containing > 80% GFP^+^ AML cells were transplanted into WT (C57BL/6) recipients.

**FIGURE 3:**
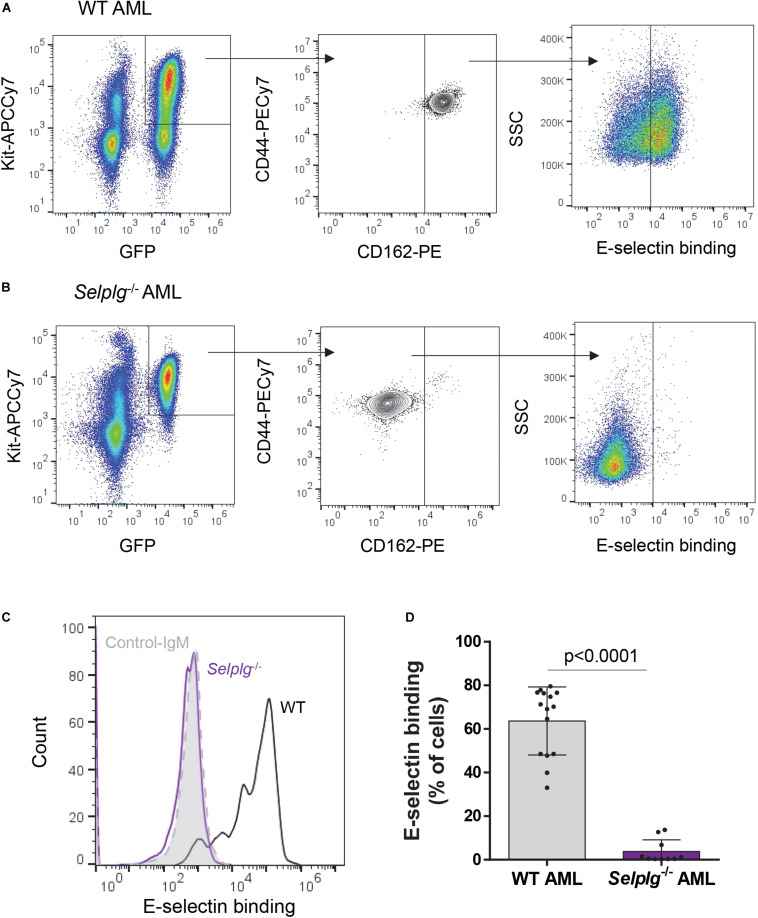
Binding of mouse KIT^+^ AML blasts to E-selectin is markedly reduced in the absence of CD162. A suspension of KIT^+^-enriched BM cells from leukemic mice (MLL-AF9 AML) was antibody-stained and incubated with a preformed complex of recombinant E-selectin–IgM and AF647-labeled antibody to IgM. **(A, B)**. Flow cytometry dot plots showing CD44/CD162 expression and E-selectin binding potential of WT **(A)** and *Selplg*^– /–^ **(B)** KIT^+^ AML cells (gated on GFP^+^ KIT^+^ population). **(C)** Overlays of E-selectin binding to gated GFP^+^ KIT^+^ AML cells. Black line, WT AML; purple line, *Selplg*^– /–^ AML; light gray filled line, control-IgM negative control. **(D)** Percentage of gated GFP^+^ KIT^+^ WT and *Selplg*^– /–^ AML blasts that bound E-selectin–IgM. *n* = 10 (*Selplg*^– /–^) or 15 (WT) mice per group (2 independent experiments); each dot represents data from one mouse. Graph shows mean ± SD. Statistical significance was calculated by unpaired two-tailed *t*-test.

We first confirmed whether the absence of CD162 (in murine *Selplg*^–/–^ AML blasts) altered E-selectin binding potential. We found absence of CD162 alone significantly reduced E-selectin binding potential of murine AML blasts when compared to WT AML blasts (from an average of 65% for WT to less than 4% for *Selplg*^–/–^ AML blasts binding to E-selectin, *p* < 0.0001) (Figures 3A–D) which was different from that observed in KG1a cells (previous Figure 1D). These data suggest that murine AML blasts may have higher reliance on CD162/*Selplg* for E-selectin binding compared to human KG1a.

**FIGURE 4:**
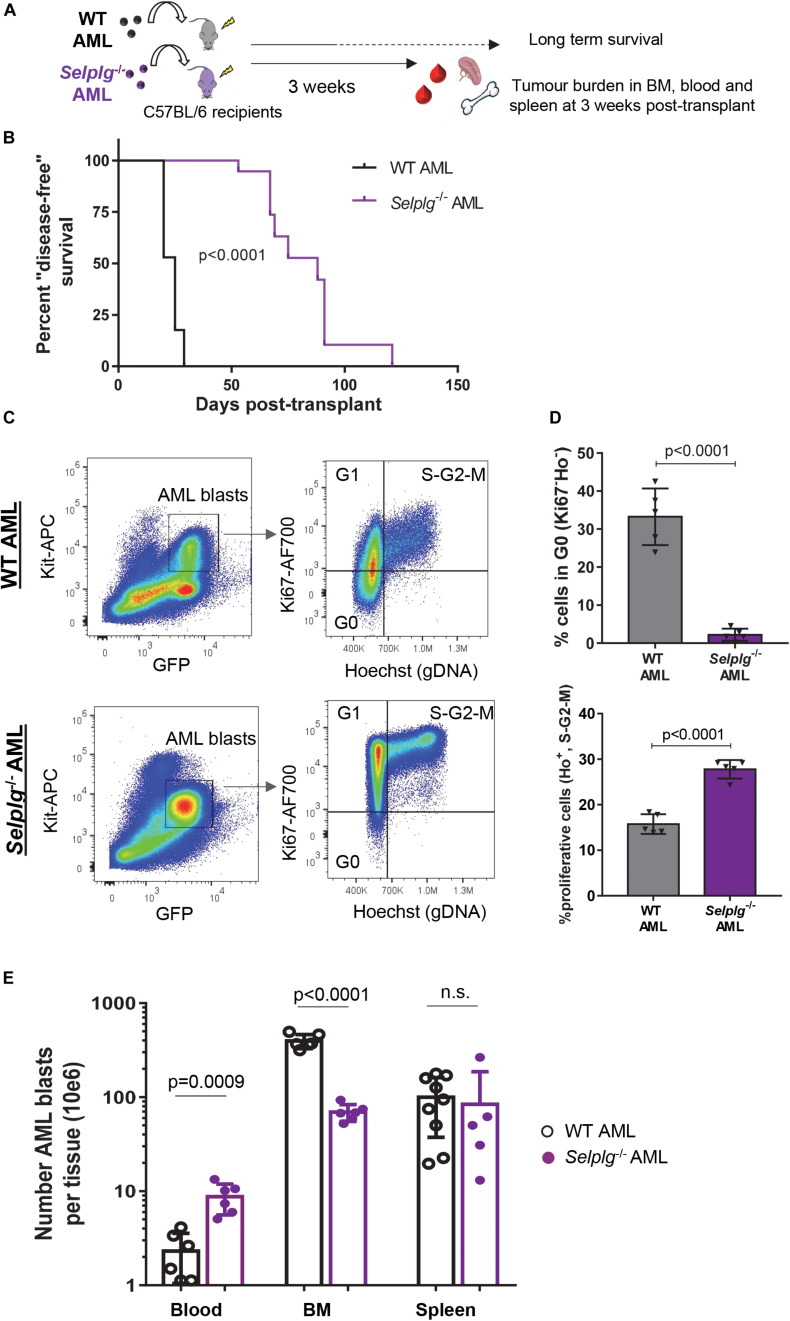
CD162 plays a key role in AML engraftment. **(A)** *Selplg*^– /–^ or WT mouse AML cells were transplanted into 4.0 Gy conditioned C57BL/6 WT recipients. Mice were evaluated for long-term survival, or euthanized at 3 weeks post-transplant for measure of BM, blood and spleen tumor burden. **(B)** Duration of “disease-free” survival. Mice were euthanized once they developed advanced leukemia, estimated according to their GFP blood levels and/or when showing signs of morbidity. After euthanasia, all mice were confirmed to have advanced leukemia with >50% GFP^+^ AML blasts in the BM. *n* = 17 recipients of *Selplg*^– /–^ AML blasts or 19 recipients of WT AML blasts, 2 independent experiments. Statistical significance was calculated by Log-Rank Mantel-Cox test. **(C)** Flow cytometry dot plots showing gating strategy for cell cycle analysis of WT (top panels) and *Selplg*^– /–^ (bottom panels) GFP^+^KIT^+^ AML blasts. The cell cycle phases were defined as G0 (Ki-67^–^, DNA = 2*n*), G1 (Ki-67^+^, DNA = 2*n*) and S-G2-M (Ki-67^+^, DNA > 2*n*). **(D)** Percentage of BM GFP^+^ KIT^+^ AML blasts in quiescence (phase G0, top histogram) and actively proliferating (phases S-G2-M, bottom histogram). Each dot represents data from an individual mouse. Shown are mean ± SD, *n* = 5 mice/group. Statistical analysis performed by unpaired two-tailed *t*-test. **(E)** Numbers of AML blasts in peripheral blood, spleen and BM were measured in mice with WT and *Selplg*^– /–^ AML. Histogram represents number of AML blasts per mouse in peripheral blood, spleen and BM. Each dot represents data from an individual mouse. All data shown as mean ± SD (*n* = 5–9 mice/group). Statistical significance was calculated by unpaired two-tailed *t*-test.

Cohorts of WT mice were transplanted with 30,000 *Selplg*^–/–^ or matching WT AML blasts to investigate potential roles for CD162 in altering leukemia progression (Figure 4A).

Although both cohorts were transplanted with the same number (30,000 BM KIT^+^GFP^+^) AML blasts each, there was a marked 3.5-fold (*p* < 0.0001) increase in median disease-free survival in recipient mice of *Selplg*^–/–^ AML compared to recipients of WT AML (Figure 4B). These data suggest that CD162 may play an important, and previously unreported, role in AML progression.

The delay in disease progression between the *Selplg*^–/–^ and WT AMLs could be explained by two main causes: the absence of CD162 expression on AML cells may lead to, (i) a slower expansion of AML cells in the host, and/or (ii) a defect in BM retention/homing potential. To investigate the first question (does AML *Selplg* expression alter proliferation potential), we performed cell cycle analysis (Ki-67 and Hoechst33342 staining) in cohorts of wildtype mice bearing *Selplg*^–/–^ or WT AML (Figures 4C,D). We found that absence of CD162 on AML cell surface led to a dramatic >90% drop in the proportion of AML blasts in quiescence (G0 phase of cell cycle). Indeed, only 2.2 ± 1.6% of *Selplg*^–/–^ AML blasts were quiescent *in vivo*, compared to 33.3 ± 7.4% in WT AML (*p* < 0.0001) (Figure 4D, top panel). Consistent with this, we also observed a 45% increase in proportion of *Selplg*^–/–^ KIT^+^ blasts actively cycling (phase S-G2-M of cell cycle with genomic DNA > 2*n* by Hoechst33342 nuclear staining) compared to WT KIT^+^ AML blasts (Figure 4D, bottom panel). Together, these data indicate that CD162 plays an important role in regulation of AML cell cycling – specifically that CD162/*Selplg* expression promotes quiescence in AML *in vivo*. Thus, the slower disease progression we observe in *Selplg*^–/–^ AML hosts is not due to a slower rate of AML cell proliferation.

To better understand the mechanisms behind the delay in leukemia progression observed with *Selplg*^–/–^ AML, a parallel cohort of mice was sacrificed at 3 weeks post-transplant for analysis of the body distribution of AML blasts [characterization of the total number of AML blasts in the BM, blood and spleen of a leukemic mouse as described previously (Winkler et al., 2013)]. To our surprise, although recipients of WT AML showed high whole BM leukemic burden as anticipated from our previous studies (Barbier et al., 2020) (396.3 ± 68.26 million AML blasts), there were six-fold less BM AML blasts in recipients of *Selplg*^–/–^ AML (69.3 ± 14.07 million AML blasts) (*p* < 0.0001, Figure 4E). In addition, a higher level of circulating AML blasts in whole blood was also observed in hosts of *Selplg*^–/–^ AML (8.8 ± 3.15 million circulating AML blasts per mouse) compared to hosts of WT AML (2.3 ± 1.26 million circulating AML blasts) (*p* = 0.0009, Figure 4E). These data would be consistent with a CD162-mediated defect in BM retention (or homing) of AML cells, resulting in an increased proportion of circulating AML blasts in the peripheral blood. It is important to note that the tumor burden in the spleen remained similar in both groups of mice transplanted with WT or *Selplg* ^–/–^ AML (Figure 4E), confirming that the low tumor burden observed in the BM was not due to a relocation of AML blasts to the spleen instead. Altogether, these data strongly suggest that *Selplg*^–/–^ AML cells have both a pronounced defect in BM homing or retention and significantly increased cell cycle kinetics.

### Deletion of CD162 in AML Sensitizes Leukemia Regenerating Cells to Therapy

**FIGURE 5:**
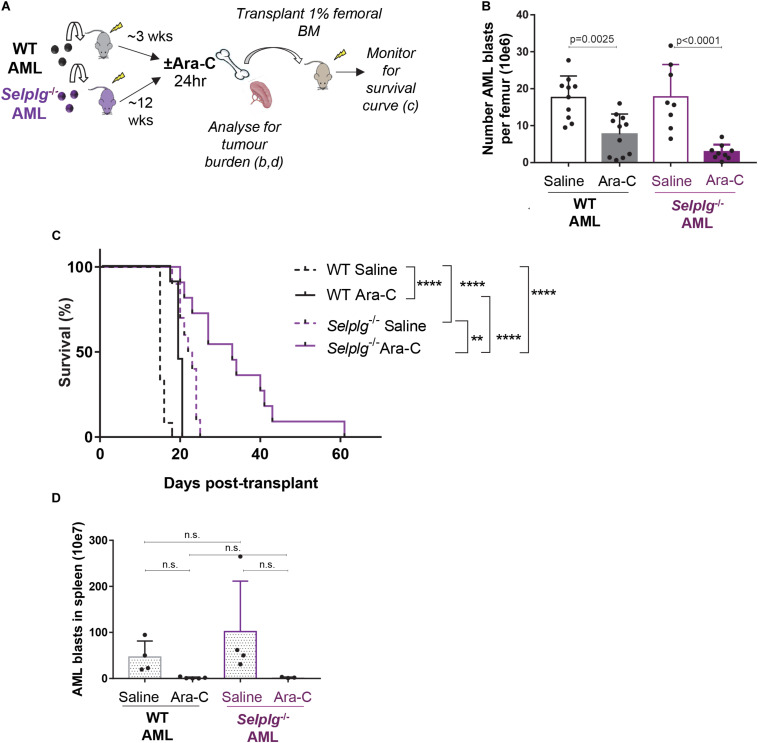
CD162 plays a key role in AML resistance to chemotherapy. **(A)** *Selplg*^– /–^or WT AML cells were transplanted into 4.0 Gy conditioned C57BL/6 WT recipients (*n* = 12 mice/group). When leukemia was fully developed (>50% GFP^+^ AML cells in BM), mice were treated with high-dose cytarabine (Ara-C) 900 mg/kg bi-daily i.p. or saline and euthanized 24 h after the first injection. Femoral BM cells were collected and analyzed for tumor burden, and 1% of the femoral content was transplanted into 4 Gy conditioned recipients for a survival experiment. **(B)** Number of GFP^+^ KIT^+^ AML blasts per femur 24 h after high dose cytarabine treatment. Data from 3 independent experiments, *n* = 17 recipients of *Selplg*^– /–^ AML and *n* = 19 recipients of WT AML per group. Statistical significance was calculated by one-way ANOVA with Dunnett’s correction for multiple comparison. **(C)** Mouse survival curve. Development of leukemia was monitored by regular bleeds; mice were euthanized when showing signs of morbidity associated with leukemia (>20% of the myeloid blood cells were GFP^+^ and high score for morbidity). Statistical significance was calculated by Log-Rank Mantel-Cox test. **p = 0.0021; ****p < 0.0001. **(D)** Number of GFP^+^ KIT^+^ AML blasts per spleen 24 h after cytarabine treatment. *n* = 7 recipients of *Selplg*^– /–^ AML blasts and *n* = 9 recipients of WT AML blasts per group. Statistical significance was calculated by one-way ANOVA with Dunnett’s correction for multiple comparison.

Loss of AML blast retention in the BM and increased cell proliferation may both contribute to an increase in AML chemo-sensitivity. To determine whether CD162 plays a role in AML chemo-sensitivity, we repeated a transplant of WT or *Selplg*^–/–^ MLL-AF9 AML blasts in wildtype hosts and investigated whether *Selplg*^–/–^ AML blasts were more sensitive to chemotherapy compared to WT AML *in vivo*. In this case, the large difference in BM AML burden between recipients of *Selplg*^–/–^ AML and WT AML (Figure 4E) necessitated an adaptation to the experimental design so that both cohorts displayed similar BM AML burden at the commencement of chemotherapy. To this aim, the same number of AML blasts (30,000 WT or *Selplg*^–/–^ BM KIT^+^GFP^+^ cells) were transplanted into wildtype mice on the same day, but cytarabine treatment started 3 weeks post-transplant for mice recipients of WT AML and at 12 weeks post-transplant in mice that received *Selplg*^–/–^ AML (Figure 5A). This resulted in a similar average AML burden in the BM of ∼50% GFP^+^ KIT^+^ AML blasts in both groups at the commencement of cytarabine treatment (Supplementary Figure 3).

Only a small proportion of leukemic blasts have the functional capacity to generate leukemia *in vivo*. These aredefined as leukemia repopulating cells (LRCs). Importantly disease will only relapse if these rare LRCs have survived treatment (Boyd et al., 2018). To determine whether LRCs in the BM of mice with WT or *Selplg*^–/–^ AML had a similar potential to survive chemotherapy and initiate relapse *in vivo*, we used a modified version of the limiting-dilution transplant assay typically used as gold-standard for quantifying functional HSC numbers (Purton and Scadden, 2007). Our adaption of this assay was previously used to demonstrate that adhesion to E-selectin in the BM vascular niche protected LRC from chemotherapy, enabling relapse to occur (Barbier et al., 2020). In this study we investigate whether the absence of selectin receptor CD162 alone could similarly reduce therapy resistance and improve duration of disease-free survival.

Cohorts of mice with advanced WT or *Selplg*^–/–^ AML (∼50% GFP^+^KIT^+^ BM AML blasts in both groups) were administered 24 h high-dose cytarabine or saline vehicle control. Femoral BM cells were then collected for; (i) analysis of residual (surviving) BM AML burden and (ii) transplant of 1% of the femoral content into cohorts of wildtype recipient mice, that were then monitored for duration of survival and AML relapse (Figure 5A).

At 24 h post-chemotherapy, the number of WT AML blasts in the BM was reduced by three-fold, while *Selplg*^–/–^ AML blast burden had reduced by seven-fold – when compared to matched no-chemotherapy (saline-treated) leukemic mice. Although this difference was not significant by ANOVA, the side by side difference between WT and *Selplg*^–/–^ AML after cytarabine treatment was significant by two-tailed *t*-test (*p* = 0.0230). These data suggest *Selplg*^–/–^ AML blasts are significantly more chemo-sensitive than WT AML (Figure 5B). This was also reflected in a significant delay in duration to leukemia “relapse” in recipients of cytarabine-treated *Selplg*^–/–^ AML BM, compared to matched WT AML (Figure 5C). In the recipients of WT AML BM, the 24 h cytarabine treatment extended median survival by 20% (4 days over matched no chemotherapy controls). In recipients of *Selplg*^–/–^AML BM, cytarabine treatment extended median survival by a further 30% (9.5 days) when compared to matched vehicle treated control mice, or 55% (17 days) when compared to saline-treated recipients of WT AML. Interestingly, the spleen tumor burden was strongly and similarly reduced in both recipients of WT and *Selplg*^–/–^ AML at 24 h post-chemotherapy, suggesting that spleen may not be a site where CD162-induced AML blast chemoresistance occurs (Figure 5D).

Finally, recipients of *Selplg*^–/–^ AML from untreated (saline-injected) donors showed a delayed leukemia onset compared to matching saline-injected WT AML, with a median survival of 22.5 days compared to 15 days, respectively (Figure 5C), despite a similar donor tumor burden in both groups. This suggests again that absence of CD162 leads to a decreased ability of AML cells to engraft in the host BM, which complements our data in Figure 4, and that absence of CD162 in AML leads to deficiencies in BM homing and/or retention. Overall, these data reveal that CD162 on AML blasts is a key cell surface receptor involved in AML niche retention, therapy resistance and relapse potential in this preclinical model.

## Discussion

This study explores the mechanisms by which vascular E-selectin-mediated adhesion promotes chemo-resistance in AML and investigate canonical cell surface receptors potentially involved. We demonstrate that CD162 is a key E-selectin receptor involved in AML progression and resistance to chemotherapy *in vitro* and *in vivo*. Mice transplanted with CD162 deleted (*Selplg*^–/–^) AML driven by the MLL-AF9 fusion oncogene have delayed leukemia progression compared to WT AML driven by the same fusion oncogene. *Selplg*^–/–^ AML cells also have reduced retention within and/or homing to the BM. In addition, we show that absence of CD162 from AML led to a marked loss in AML blast quiescence and a strong increase in cycling AML in the BM. This was associated with 2.5-fold increased chemo-sensitivity in *Selplg*^–/–^ AML blasts compared to WT AML *in vivo*. These findings may be due to the direct effect of interactions between CD162 and E-selectin, as in our *in vitro* model we found that E-selectin-mediated chemo-resistance was abrogated when CD162 receptor was absent from the human CD34^+^ AML cell line KG1a.

Interestingly, KG1a cell adhesion to P-selectin (to which CD162 also binds) did not appear to mediate their chemo-resistance *in vitro* (Figure 2A) similar to our previous observation with MLL-AF9-driven mouse AML cells (Barbier et al., 2020). This suggests that CD162 binding to E-selectin is unique in specifically inducing chemo-resistance signaling in AML blasts and LRCs.

Our findings also highlight a key difference between AML cell response to E-selectin and that of endogenous HSCs. Using *Selplg*^–/–^ mice we have previously shown that CD162 was redundant for E-selectin-mediated adhesion of murine HSCs (Winkler et al., 2004, 2012); in contrast we now report that CD162 is critical for adhesion and binding of murine AML blasts to E-selectin. Similarly, we show herein that CD162 plays an important functional role in AML cell biology, while absence of CD162 from HSC cell surface did not affect their *in vivo* proliferation nor their *in vitro* response to E-selectin (Winkler et al., 2004, 2012). Together, these data demonstrate that the receptors involved, and cellular response to E-selectin-adhesion may differ between non-malignant HSCs and malignant AML cells. This may be due to differences in cell surface receptor glycosylation associated with oncogenesis, as altered cell surface glycosylation, which may include the generation of *de novo* E-selectin-binding potential, is one of the hallmarks of malignant transformation (Hakomori, 1996).

There is increasing evidence that CD162 may play a role in cancer cell interactions with their environment (Dimitroff et al., 2005; Raes et al., 2007; Zheng et al., 2013; Heidemann et al., 2014; Tinoco et al., 2016; Li et al., 2017; Spertini et al., 2019). To date, few studies have addressed the role of CD162 in hematological malignancies, and most of them have focused on CD162 as a P-selectin ligand rather than an E-selectin ligand. In neutrophils, CD162 is known as a major ligand for P-selectin, but is not as important for E-selectin as E-selectin has other ligands as well. Indeed, loss of CD162 from murine neutrophils leads to strongly impaired rolling on P-selectin, while the few neutrophils that still tether to E-selectin retain their normal velocities (Xia et al., 2004). These data confirm that CD162 can play different roles depending on the selectin to which it binds.

CD162 has been shown to be important for chronic myelogenous leukemia (CML) engraftment (Krause et al., 2014). This may involve homing-type interactions with E-selectin and/or P-selectin on the BM vasculature or CML retention at BM niche. As the role of P-selectin was not excluded in these studies, it is unclear how much E-selectin may be involved (Krause et al., 2014). Another study showed that CD162 expressed by multiple myeloma cells was involved in their growth, dissemination, and drug resistance, however, this was through interaction with P-selectin, not E-selectin (Azab et al., 2012). Blocking of P-selectin or its CD162 binding site with neutralizing monoclonal antibodies increased the sensitivity of multiple myeloma cells to bortezomib (Muz et al., 2015). However, a role for CD162 binding to E-selectin was not investigated and cannot be excluded from this study.

Our study is the first evidence of a direct effect of CD162 in mediating resistance of AML cells to chemotherapy, and our *in vitro* data suggests that this effect may specifically involve the interaction of CD162 with E-selectin, not P-selectin. Further studies are required to confirm a direct role of CD162/E-selectin interactions in mediating AML progression and chemoresistance.

It is worth noting that the role of CD162 on AML physiopathology may differ in different human or murine cell lines. Indeed, AML is a highly heterogeneous disease, that can be caused by variety of chromosomic rearrangements with translocations leading to production of chimeric proteins (such as AML1-ETO or MLL fusion proteins) and/or somatic mutations such as FLT3-ITD or NMP1 (De Kouchkovsky and Abdul-Hay, 2016). While the mouse model of AML used in our studies mimics 11q23-translocations involving the MLL gene rearrangement and fusion, the human AML cell line KG1a exhibits a complex karyotype with several structural aberrations, including FGFR1OP2-FGFR1 gene fusion, NRAS^G12D^ mutation and TP53^c.672+1G→A^ splice donor mutation, but no translocation involving either MLL or AML1 genes (Mrózek et al., 2003). Studies from Spertini et al. (2019) have shown that CD162 was highly expressed in most primary acute leukemia cells tested (from over 90 patients), but its role in AML blasts’ interaction with E-selectin was variable. Therefore, it is likely that the role of CD162 may differ depending on the genetic profile of the leukemia and on patient characteristics. Whether the results presented in this manuscript may apply to all types or only to certain subgroups of AMLs, remains to be explored.

Interestingly, despite our findings suggesting a major role for CD162 in mediating E-selectin-induced chemo-resistance, we found that absence of CD162 from human AML cell line KG1a did not significantly reduce E-selectin adhesion. Absence of CD44 from KG1a also did not reduce their binding potential to E-selectin, but deletion of both CD162 and CD44 did result in a significant ∼60% reduction in E-selectin adhesion potential. A recent study also confirmed that inhibition of either CD162 or CD44 on human monocytic leukemia U937 cell line did not alter rolling on E-selectin, whereas knockdown of both CD162 and CD44 increased rolling velocity and reduced U937 adhesion to immobilized E-selectin under flow conditions by about 40% (Spertini et al., 2019). Collectively, these data suggest that apart from CD162 and CD44, additional receptors are contributing to E-selectin-mediated adhesion and potentially remain to be characterized. It is also possible that the receptors that mediate AML adhesion under blood flow (tethering) may be distinct from those mediating E-selectin-induced chemo-resistance signaling (in situations where cells are in close contact with the endothelium, such as when lodged in the BM vascular niche).

In the current study we also report no major role for CD44 in E-selectin-mediated chemo-resistance *in vitro*. This was unexpected as CD44 has been extensively studied for roles in tumorigenesis (Morath et al., 2016), metastasis (Orian-Rousseau, 2010), and chemo-resistance (Wang et al., 2014; Xu et al., 2015), in solid tumors as well as hematological malignancies (Thapa and Wilson, 2016). We attempted to generate MLL-AF9 AML from mouse *Cd44*^–/–^ HSCs for confirmation. However, all four of our attempts failed and we were unable to generate any serially transplantable transduced *Cd44*^–/–^ AML from murine fetal liver HSPCs, even in situations where parallel transductions on HSPCs from *Selplg*^–/–^ fetal liver were successful at generating AML. These observations suggest expression of CD44 may be critical in the process of malignant transformation or self-renewal, and are congruent with the recent observation that CD44 is crucial for CML engraftment in mouse models (Krause et al., 2006). Anti-CD44 antibodies have been trialed in pre-clinical and clinical studies, and were shown to eradicate AML stem cells in mouse models (Jin et al., 2006), although their clinical activity so far is very limited (Vey et al., 2016). CD44 has over 40 isoforms and splicing variants, many of which bind other components of the niche such as hyaluronic acid and heparin (Ghaffari et al., 1999; Morath et al., 2016). Only one specific glycoform of one of these CD44 splice forms (HCELL) can bind to E-selectin. High expression of some CD44 splice forms have been reported to associate with a poor prognosis and shorter survival in AML patients, although it remains unclear the CD44 ligands involved (Legras et al., 1998; Thapa and Wilson, 2016).

Overall, although there is a large amount of literature on CD44 in cancer pathophysiology, the specific role of CD44 interaction with E-selectin has been poorly studied *in vivo*. In our *in vitro* studies, we found that E-selectin adhesion still promoted significant chemo-resistance in an AML cell line even when *CD44* had been deleted, suggesting that CD44 may play only minor, or no role in E-selectin-mediated AML chemo-resistance.

In conclusion, our studies have identified CD162 as a key cell surface receptor mediating AML development and resistance to cytotoxic chemotherapy. Blocking the binding of CD162 to E-selectin represents a new potential therapeutic target to delay disease progression, sensitize AML LRCs to chemotherapy and improve overall therapeutic outcomes.

## Data Availability Statement

The raw data supporting the conclusions of this article will be made available by the authors, without undue reservation.

## Ethics Statement

The animal study was reviewed and approved by Animal Experimentation Ethics Committee of the University of Queensland.

## Author Contributions

JE designed the research, performed the experiments, analyzed the data, generated the figures, and wrote the manuscript. JT helped with design of some experiments, analyzed some data, designed the sgRNA for CRISPR gene editing, and commented on the research direction. VB performed experiments and helped with design of some experiments. J-PL participated in the design of the research, commented on research direction, helped analyze some of the data, and critically edited manuscript. IW initiated and supervised the research, analyzed the data, and critically edited manuscript. All authors have discussed and commented on the manuscript.

## Conflict of Interest

The authors declare that the research was conducted in the absence of any commercial or financial relationships that could be construed as a potential conflict of interest.

## Footnotes

1 https://www.deskgen.com
