# Supplementary material for: Acute Myeloid Leukemia Chemo-Resistance Is Mediated by E-selectin Receptor CD162 in Bone Marrow Niches
Source: Front Cell Dev Biol. 2020 Jul 24;8:668. doi: 10.3389/fcell.2020.00668 (PMC7393995; doi:10.3389/fcell.2020.00668)
Supplement: Supplementary file 1 [file Data_Sheet_1.PDF]

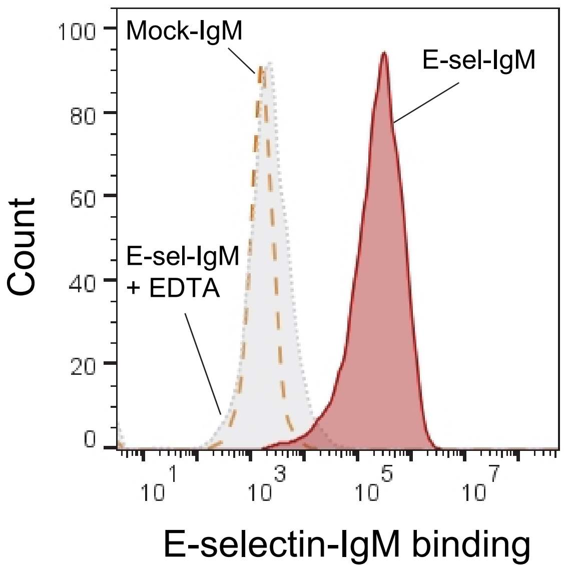

Supplementary Figure 1. E-selectin binding is abrogated with calcium-chelating EDTA. WT KG1a cells were incubated with a preformed complex of E-selectin–IgM (or matching empty vector) and AF647-labeled antibody to IgM, ± 15mM EDTA. Graph shows overlays of E-selectin binding to gated singlets/live cells. Filled dotted grey line: EDTA negative control; brown dotted line: Mock-IgM negative control; filled red line: E-selectin-IgM.

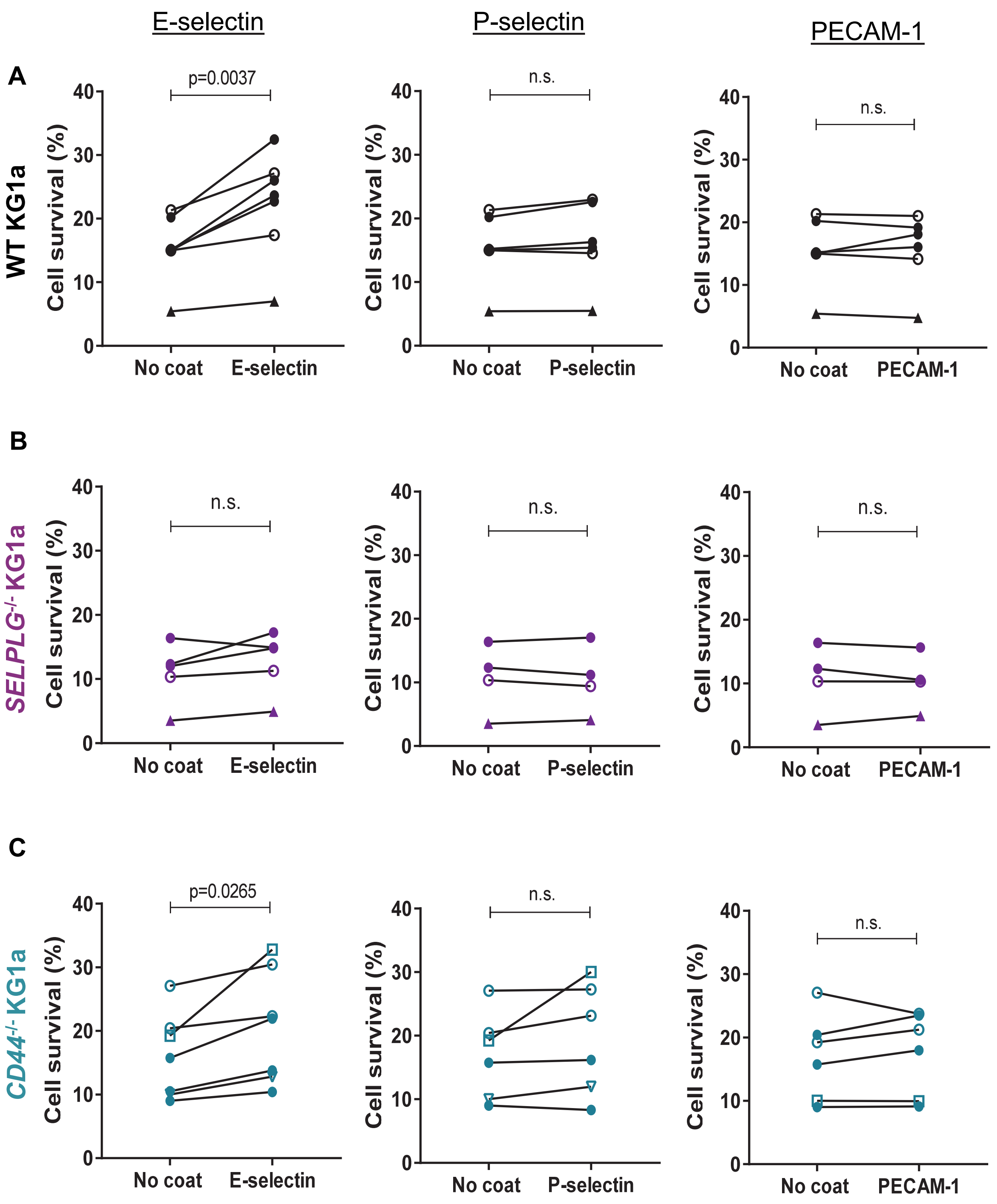

Supplementary Figure 2. (**A**) WT, (**B**) *SELPLG*$^{-/-}$ and (**C**) *CD44*$^{-/-}$ KG1a cells were immobilized on recombinant adhesion molecules: E-selectin (left panels), P-selectin (middle panels) and PECAM-1 (right panels) and treated with 10μg/mL cytarabine. Cell viability was assessed after 48h of incubation at 37ºC. The percentage of surviving cells was calculated in comparison to untreated cells seeded at the same density and in the same conditions. Several individual clones (derived from single-cell sorting after gene editing) were tested in this assay. Represented are paired experiments; each paired set of dots represents data from a different clone, and each dot represents the average data from 5 replicate wells. Statistical significance was calculated by two-tailed paired t-test.

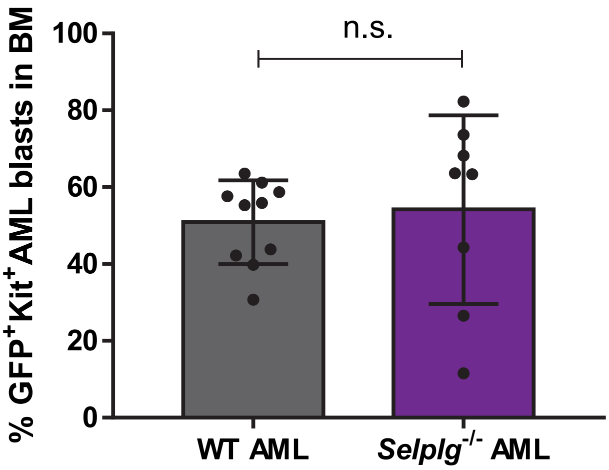

Supplementary Figure 3. WT or *Selplg*$^{-/-}$ mouse AML cells were transplanted into 4Gy conditioned C57Bl6 WT recipients (n= 10 and 8 mice/group, respectively). Mice were euthanised at 3 weeks (WT AML) or 12 weeks (*Selplg*$^{-/-}$ AML) post-transplant. Graph shows tumour burden (% GFP^{+}Kit^{+} AML blasts) in femoral BM. Data shown as mean ± S.D. Statistical significance was determined by two-tailed unpaired t-test.

Antibodies	Clones	Suppliers	Dilutions
Anti-human			
CD162-BV421	KPL-1	BioLegend	1/100
CD162-PE	KPL-1	BioLegend	1/200
IgM-AF647	Polyclonal	Jackson ImmunoResearch	N/A
Anti-mouse and human			
CD44-PECy7	IM7	BioLegend	1/300
CD44-APC	IM7	BioLegend	1/400
Ki67-AF700	B56	BD Pharmingen	1/20
Anti-mouse			
CD162-PE	29H1	BD Pharmingen	1/200
Kit(CD117)-APCCy7	2B8	BioLegend	1/200
Kit(CD117)-APC	2B8	BioLegend	1/200
CD45.2-FITC	104	BioLegend	1/200

Supplementary table 1. Antibodies used for flow cytometry
